# Development of a nondiapausing strain of northern corn rootworm with rearing techniques for both diapausing and nondiapausing strains

**DOI:** 10.1038/s41598-021-97452-8

**Published:** 2021-09-09

**Authors:** Man P. Huynh, Chad Nielson, B. Wade French, Dalton C. Ludwick, Ryan W. Geisert, Adriano E. Pereira, Julie Barry, Lisa N. Meihls, Sharon K. Schneider, Bruce E. Hibbard

**Affiliations:** 1 Division of Plant Science & Technology, University of Missouri, Columbia, MO USA; 2 Department of Plant Protection, Can Tho University, Can Tho, Vietnam; 3 North Central Agricultural Research Laboratory, USDA-Agricultural Research Service, Brookings, SD USA; 4 Department of Entomology, Texas A&M University AgriLife Extension, College Station, TX USA; 5 Plant Genetics Research Unit, USDA-Agricultural Research Service, Columbia, MO USA; 6 Bayer Crop Science, Union City, TN USA

**Keywords:** Entomology, Model invertebrates

## Abstract

The northern corn rootworm, *Diabrotica barberi* Smith & Lawrence, has a univoltine life cycle that typically produces one generation a year. When rearing the northern corn rootworm in the laboratory, in order to break diapause, it is necessary to expose eggs to a five month cold period before raising the temperature. By selective breeding of the small fraction of eggs that hatched without cold within 19–32 days post oviposition, we were able to develop a non-diapausing colony of the northern corn rootworm within five generations of selection. Through selection, the percentages of adult emergence from egg hatch without exposure to cold treatment significantly increased from 0.52% ± 0.07 at generation zero to 29.0% ± 2.47 at generation eight. During this process, we developed an improved method for laboratory rearing of both the newly developed non-diapausing strain as well as the diapausing strain. The development of the non-diapausing colony along with the improvements to the rearing system will allow researchers to produce up to six generations of the northern corn rootworm per year, which would facilitate research and advance our knowledge of this pest at an accelerated rate.

## Introduction

The northern corn rootworm, *Diabrotica barberi* Smith & Lawrence, and western corn rootworm *Diabrotica virgifera virgifera* LeConte, are responsible for approximately 2 billion USD in yield losses and management costs annually for U.S. corn growers^1^. This is primarily due to the damage caused by the larval stages that feed on the roots of corn (*Zea mays* L.) and results in a reduction in the ability of corn plants to uptake water and nutrients from the soil. With wind, rain, and at least moderate feeding damage, these plants often topple over or lodge in the field^2,3^. Lodged plants are lower yielding due to less light capture and are also more difficult to pick up with mechanical harvesters, causing an additional yield loss component due to unharvested grain^4^. There have been several strategies developed to manage both northern and western corn rootworms (e.g., chemical insecticides, transgenic plants, and cultural control techniques such as crop rotation), but both species have evolved mechanisms to circumvent these strategies. For example, both the northern and western corn rootworms have evolved resistance to crop rotation^5–7^, chemical insecticides^8–12^, and transgenic maize hybrids expressing insecticidal crystalline toxins from *Bacillus thuringiensis* (Bt) Berliner, developed for their control^13–21^.

In general, both the northern and western corn rootworms are univoltine with one generation per year and overwinter as eggs in the soil^22,23^. The northern corn rootworm has evolved resistance to crop rotation through selection for an extended diapause trait, where eggs hatch after two or more winters as opposed to just one winter^5,24–26^. Because of the long obligate and diapause phase of rootworm, laboratory studies with wild populations are difficult and lengthy. To combat this, a non-diapausing colony of the western corn rootworm was developed in the mid-1970s^27^. The non-diapausing laboratory colony requires no cold treatment of eggs, enabling the production of approximately four to six generations per year rather than a single generation.

The availability of a non-diapausing western corn rootworm colony has been documented to be valuable. Since its selection^27^, excluding experiments with field-collected beetles, nearly all laboratory and greenhouse research for the western corn rootworm have utilized this single population or crosses of it. In fact, without the availability of the non-diapausing western corn rootworm, selection experiments to determine mechanisms of resistance development of the western corn rootworm to Bt traits would not be possible^28–32^. In 2008, laboratory studies related to resistance development to the Bt trait Cry3Bb1 were conducted with non-diapausing western corn rootworm. The studies demonstrated that resistance development to the trait can occur after only three generations of continuous exposure^30^. Soon after this, resistance to the Cry3Bb1 trait was found in continuous corn fields utilizing the trait year after year. The fields in question had been planted with the trait for 3 years, showing a similar resistance development path to the laboratory assays performed just years prior^13^. Nonetheless, a non-diapausing northern corn rootworm colony is not yet publicly available. Therefore, developing and utilizing the non-diapausing colony for northern corn rootworm would give researchers a head start on the development of resistance management strategies involving this important pest.

Compared to the western corn rootworm, there is not much information related to the rearing of the northern corn rootworm. In Geisert and Meinke^33^, eggs were collected from gravid northern corn rootworm females from corn fields across Nebraska and reared over several generations in order to determine the rate of extended diapause present in each population. For their studies, eggs were maintained at 22 °C for 1 to 2 months after initial oviposition, then 10 °C for approximately 30 days, followed by 5 °C for approximately 6 months, finally the eggs were moved to 22 °C to promote the eclosion of neonate larvae and eventual egg hatch^33^. This combination of temperatures and exposure times were necessary to facilitate prediapause development, diapause termination, and finally post diapause development. The process is lengthy and tedious and does not lend itself well to most laboratory research.

For the current study, our primary goal was to develop a non-diapausing colony of the northern corn rootworm by selectively breeding beetles from larvae that hatched within 19–32 days post oviposition. Additionally, since methods for mass rearing northern corn rootworm have not been previously published, we establish full rearing methods for both the non-diapausing strain and diapausing strain of the northern corn rootworm. The development of the non-diapausing colony combined with robust rearing methods will facilitate research and advance our knowledge of this important pest at an accelerated rate.

## Results

**Figure 1:**
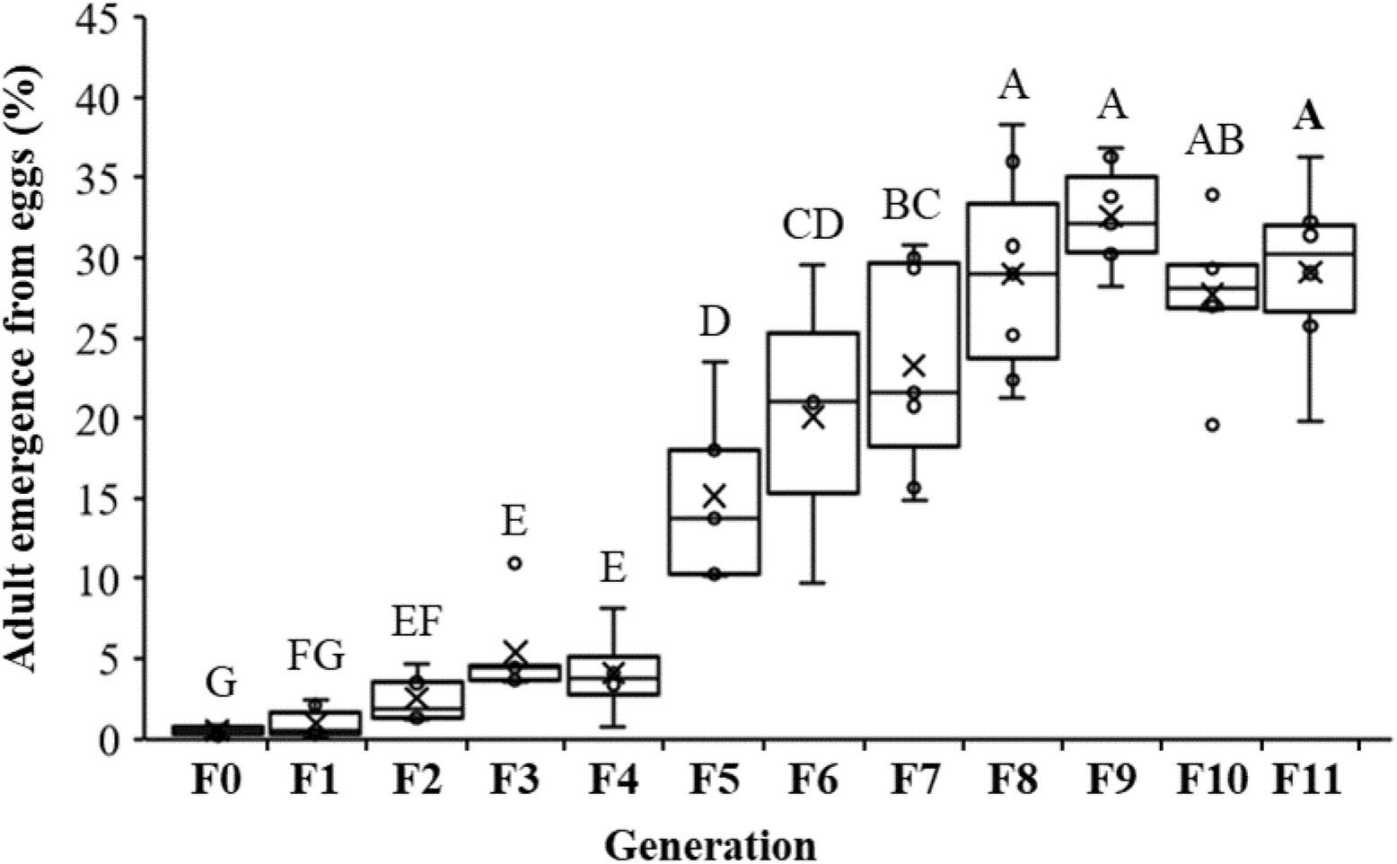
Percentages of adult emergence from egg hatch within 26–32 days post oviposition. Tukey’s box plots with median (black line), the 25th and 75th percentiles (bottom and top of box, respectively), and the 5th and 95th percentiles (whiskers) are shown. Boxes with different letters are significantly different (*P* < 0.05).

Through the use of selective breeding of the northern corn rootworm eggs that hatched within 19–32 days post oviposition without cold exposure for 11 generations, the percentage of adult emergence from eggs was significantly increased (*P* < 0.0001, F_11,50_ = 79.88, Fig. 1). Prior to initiating selection, percent adult emergence from egg hatch within 19–32 days post oviposition was 0.52 ± 0.07% (4341 adults returned from ~ 844,800 eggs). By generation five of the selection, percent adult emergence from eggs within 19–32 days of oviposition had significantly increased to 15.15 ± 0.03%, which is a ~ 29-fold increase compared with prior to selection. The maintenance level of the non-diapausing colony, which is the number of adults that produces enough eggs needed to sustain the population, was reached at generation five. After the fifth generation of selection, the percentage of adult emergence from eggs continuously increased to 29.0 ± 2.47% at generation eight and remained at that level through generation eleven (Fig. 1).

**Figure 2:**
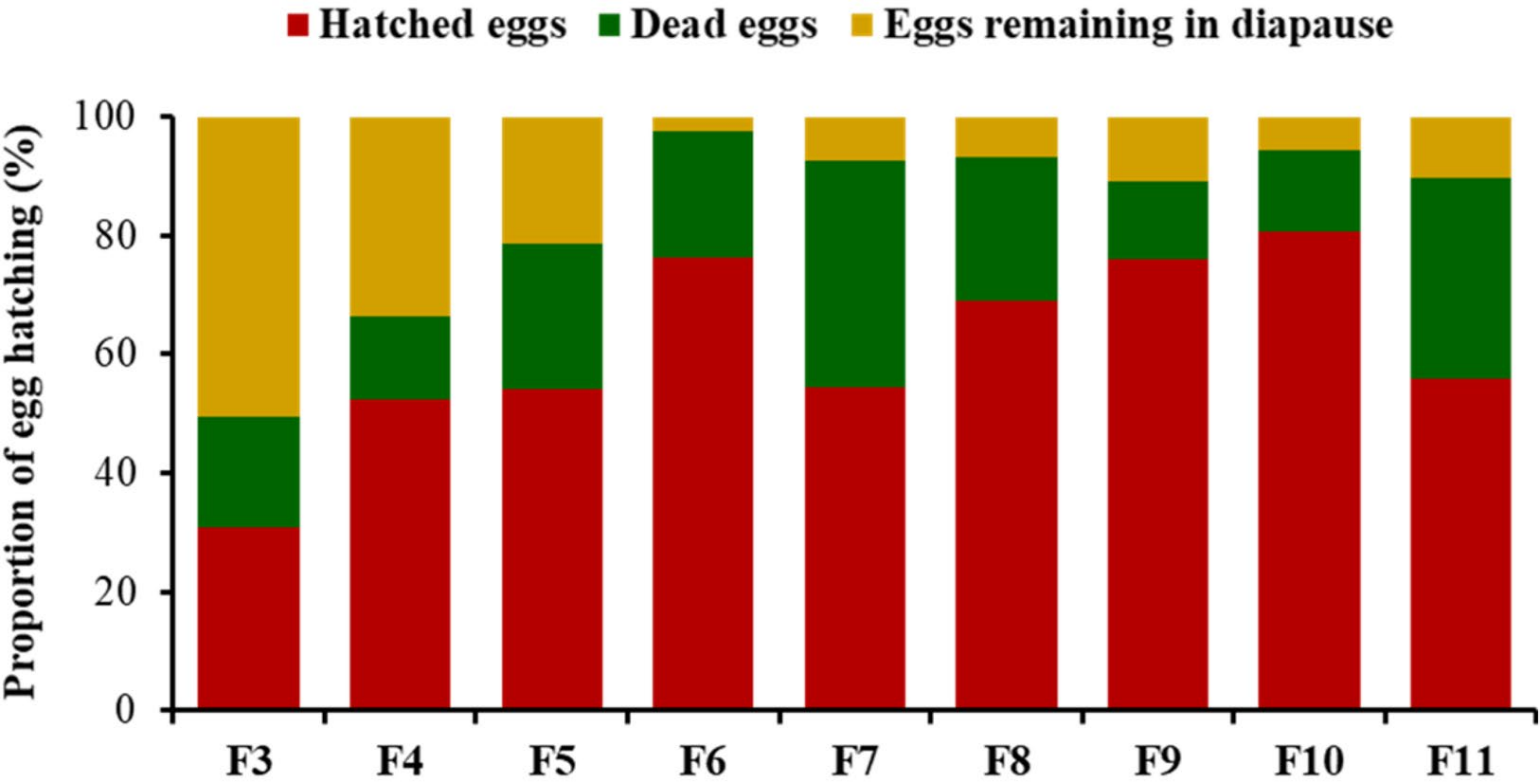
Percentages of hatched eggs, dead eggs, and eggs remaining in diapause for multiple generations.

Egg viability (i.e., hatched, dead, and diapausing egg numbers) was determined from generation three to generation eleven of the selection. At generation three, percent egg hatch was 30.7 ± 2.33% (Fig. 2). The selection resulted in significant increases in the percentage of egg hatch to 81.7 ± 1.76% (F10) compared with generation three (*P* < 0.0001, F_8, 19_ = 14.29). Percentage of eggs remaining in diapause was continuously reduced from 50.7 ± 3.18% (F3) to 2.3 ± 0.88% (F6) (*P* < 0.0001, F_8, 19_ = 8.15, Fig. 2). No significant difference was observed in the percent of diapausing eggs between generations six and eleven. Percentage of deceased eggs varied significantly, ranging from 14.0 ± 1.15% (F4) to 38.3 ± 6.25% (F7) (*P* = 0.0012, F_8, 19_ = 5.40, Fig. 2). More deceased eggs were observed in generations seven and eleven compared to other generations (Fig. 2).

**Figure 3:**
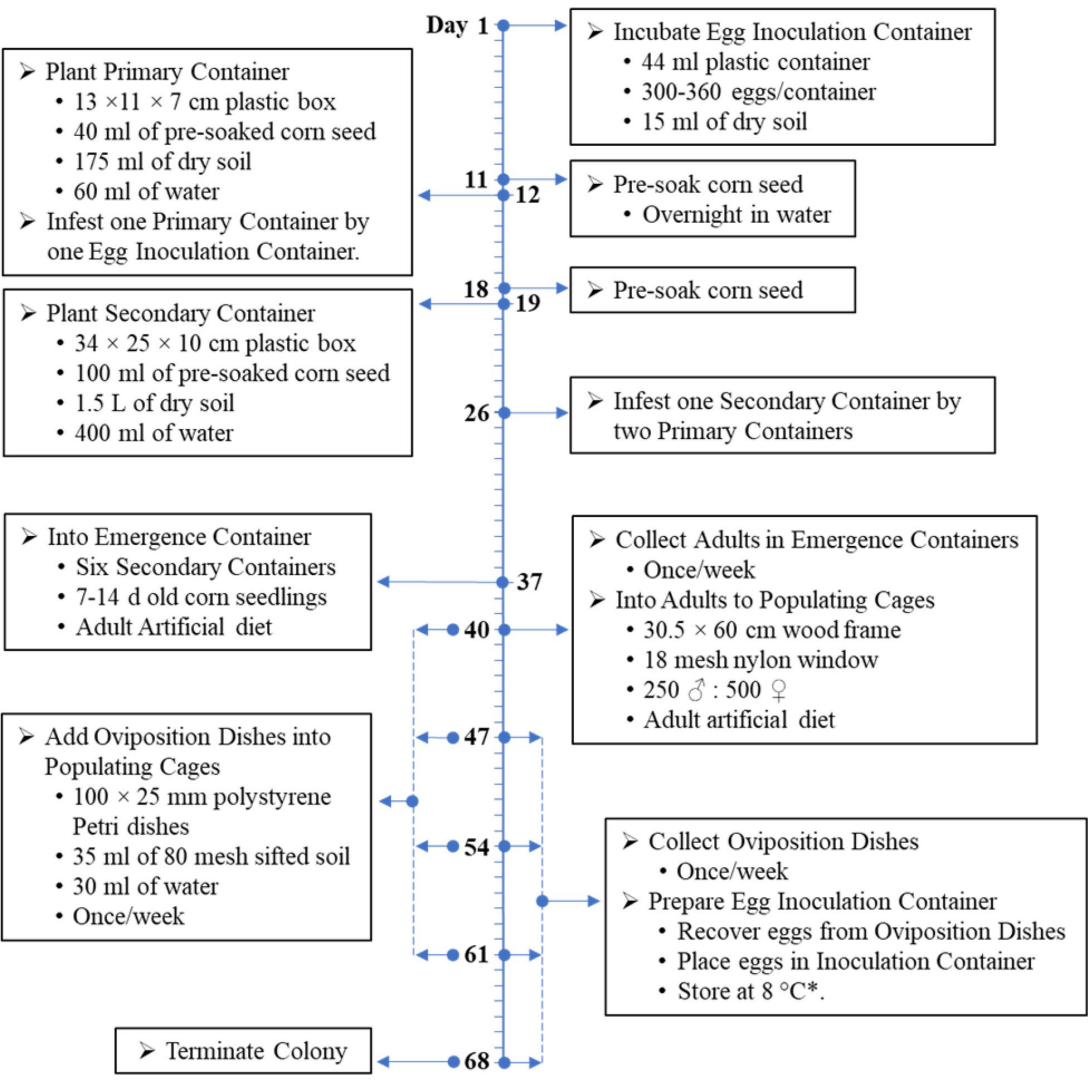
Rearing procedure for diapausing and non-diapausing strains of the northern corn rootworm under laboratory conditions. *For the diapausing strain, eggs are incubated for 14 d to allow for prediapause embryonic development prior to cold storage and then the eggs are stored for at least five months to break diapause and synchronize hatch.

The rearing and handling techniques of northern corn rootworm have been developed and improved for over 20 years. These methods consistently produce high quality of the northern corn rootworm (both diapausing and non-diapausing strains). The streamlined rearing method is shown in Fig. 3.

## Discussion

We have successfully developed a non-diapausing strain of northern corn rootworm by selecting for early egg hatch. Generation time is greater than six months for the diapausing northern corn rootworm in the laboratory because they typically undergo a five month long cold period before being moved to 25 °C for hatching. In addition to fulfilling an obligate overwintering period in their life cycle, the chilling period synchronizes egg hatch^33^. For non-diapausing egg selection, laboratory maintained diapausing populations were continually selected and reared adults from eggs that hatched within 19–32 days of oviposition without being subjected to a cold period, which drastically reduced the generation time to approximately two months. As a result, up to six generations of the selected non-diapausing colony could be produced per year, whereas the diapausing colony produces fewer than two generations per year.

Selection for egg hatch without cold exposure was previously used to develop a non-diapausing strain of the western corn rootworm^27^. Selection pressure was first applied using egg hatch within 12 weeks of oviposition at 20–22 °C, and then increasingly intensive selection pressure was applied in each generation (reduction of 1–2 weeks per generation) through generation ten. After the tenth generation, the selection for egg hatch was continued using eggs hatching within 4 weeks until the fourteenth generation. Branson^27^ reported that the non-diapausing strain of western corn rootworm was established after nine generations of selection. In comparison, the selection method used in this study was more intense as eggs were selected to 19–32 days post oviposition for multiple generations. As a result, the non-diapausing strain of northern corn rootworm was established after five generations of selection. Branson^27^ documented that time to initial hatch for the western corn rootworm non-diapausing colony was significantly reduced among all populations examined until generation eleven, while no significant difference was found between generations eleven and fourteen. A similar pattern was found in the selection of non-diapausing strain of northern corn rootworm. Our results revealed that the continued selection for egg hatch of the northern corn rootworm resulted in continuously significant increases of percent adult emergence from 0.52 ± 0.07% (F0) to 29.0 ± 2.47% (F8), a 55-fold increase, whereas no significant difference in the percent adult emergence from eggs was observed after generation eight. Further selection efforts could focus on selection for egg hatch within shorter timeframes (e.g., 19–25 days after oviposition), which could further reduce the generation time.

Egg diapause is an adaptive trait that delays development under unfavorable circumstances. Egg hatch days post oviposition have been documented to be highly variable in northern corn rootworm, ranging from 19 to 32 days (current study) to 2 years^5,7,34,35^. Compared with previous observations, the percent hatch at the initiation of selection is slightly lower which is likely due to the selection of early hatch within 19–32 days of oviposition at the first generation. Furthermore, in the early 1980s, Krysan et al.^5^ found ~ 7% of northern corn rootworm eggs exhibited non-diapause. In this study, the reduction in percent eggs remaining in diapause following several generations of selection may reveal genetic plasticity in the diapause trait of northern corn rootworm eggs, indicating that the diapause trait is quantitatively inherited allowing the northern corn rootworm to adapt to a wide range of environmental conditions. French et al.^35^ documented that the extended diapause trait of northern corn rootworm eggs where eggs require two overwintering periods to hatch (2 years) was highly heritable and strongly influenced by female genotype. The authors compared reciprocal single pair crosses among beetles from a laboratory colony with the ancestral 1-year diapause trait and field collected beetles with the 2-year extended diapause trait and reported that the 2-year extended diapause females laid significantly more extended diapause eggs compared with the females with the 1-year diapause trait, regardless of male genotype. The extended diapause trait of northern corn rootworm has been reported to have increased from ~ 0.3% in 1965^34^ to 30–40% in 1984^5^ and 14–51% in 1992^25^. Future research might focus on identifying and utilizing the gene(s) involved in regulation of the diapause trait in northern corn rootworm as a means of controlling this important insect pest.

There is one important difference between the western and northern corn rootworms that could potentially affect the ability of the non-diapausing colony to mirror resistance development in the field. Unlike the western corn rootworm, the northern corn rootworm can exhibit an extended diapause trait in the wild. Rootworms with this extended diapausing trait will remain as eggs for an additional year, and in some cases several years^25,26^. This behavior was first described by Chiang in 1965 where it was reported to only occur in 0.3% of the total population^34^. Over time, the trait prevalence has increased with rates as high as 40 to 50% of the northern corn rootworm population in select locations^5,25^. This increase in extended diapause is likely related to the frequent use of a 2-year crop rotation for the control of northern and western corn rootworms. By remaining in the soil for an additional year the rootworms are able to avoid the unfavorable conditions 1 year and hatch the next year when corn is most likely planted again. While this does not negate the importance or functionality of laboratory assays utilizing a non-diapausing colony of northern corn rootworm, it should be known that the comparisons of laboratory assays and field developed resistance may not match up as closely as those of the western corn rootworm. In theory, field populations of northern corn rootworm that hatch after more than 1 year may delay the development of resistance as they would not have experienced the same level of selective pressure as those that hatch year after year. It may be necessary to consider the level of extended diapause present in particular areas when comparing them with laboratory assays utilizing the non-diapausing colony.

Monitoring efforts for resistance development in the northern corn rootworm is also facilitated by the availability of an artificial diet capable of supporting larval growth and recent improvements in egg recovery from wild beetles. Huynh et al.^36^ developed a larval artificial diet specifically for the northern corn rootworm, which is derived from corn rootworm larval diets^37,38^. Baseline susceptibility of the northern corn rootworm to the four current Bt products targeting corn rootworms was then determined using this diet^39^. Prior to the availability of the larval optimized diet, an artificial diet for the southern corn rootworm (*Diabrotica undecimpunctata* howardi Barber)^38^ that is inferior to the optimum diet has been used to determine the susceptibility of the northern corn rootworm to Bt straits mCry3A and eCry3.1Ab^40^. Furthermore, Pereira et al.^41^ documented techniques to efficiently obtain eggs from wild northern corn rootworm females in the laboratory, with more than 47,000 northern corn rootworm eggs obtained from just 340 females from a single location. Prior to their work, obtaining eggs from wild northern corn rootworm populations to monitor for resistance to Bt crops was a challenge.

The rearing method of northern corn rootworm we describe here is reliable and is not excessively labor-intensive. Eggs are easily prepared and stored, larvae emerge in close proximity to corn roots, pupae do not need to be handled, and emerging adults can be easily collected and reared. The rearing method of northern corn rootworm was developed by adapting the method used for western corn rootworm^42^. Key differences for rearing protocols between the northern and western corn rootworms include surface preparation of the oviposition dish, the use of oviposition dish coverings that increases soil humidity and provides seclusion, and the addition of a slice of adult artificial diet to the oviposition dish covering to entice females into the oviposition dish. Northern corn rootworm beetles can also be collected weekly to reduce labor. However, the long term effect weekly collection on adult fitness has not been determined. French and Hammack^43^ also demonstrated that the northern corn rootworm pupae can be individually handled to differentiate sexually dimorphism for adult fitness studies.

The availability of a non-diapausing colony combined with the rearing system improvements that allows for the production of multiple generations of high-quality of northern corn rootworm will accelerate the growth of knowledge of this pest. In the past, the only method for observing resistance development in the northern corn rootworm was to collect beetles from problem fields and evaluate them against control products in the laboratory^19^. While this method allows for the detection of resistance within the population, it means that any solutions devised would have to work around resistance to the products already being present. With the availability of a non-diapausing colony, we are capable of producing six back-to-back generations a year, this means that laboratories can expose the insects to various products and potentially monitor in just 1 year how resistance develops over six generations. This tool would thus allow for the development of more proactive resistance management strategies. Implementing these strategies prior to commercialization would not only save growers from unexpected damages but also prolong the usefulness of said products against the northern corn rootworm.

## Materials and methods

### Selection for non-diapausing trait

A northern corn rootworm diapausing colony was initiated at the USDA-ARS Northern Grain Insects Research Laboratory in Brookings, SD, in 1996. In May 2018, a subset of this colony was used to initiate the selection for the non-diapause trait. This laboratory maintained diapausing population of the northern corn rootworm in Brookings, SD undergoes a five month long cold period before being moved to 25 °C for hatching. This chilling period facilitates egg hatch synchrony^33^. Preliminary observations indicated that a small subset of larvae emerged from diapausing eggs incubated for 4 weeks at 25 °C and 60% relative humidity (RH).

To start the development of a non-diapausing colony, laboratory maintained diapausing populations were continually selected for egg hatch within 19–32 days post oviposition without being subjected to a cold period. Only larvae from eggs that hatched within these time frames were reared to adulthood using a northern corn rootworm specific rearing procedure described below. Briefly, oviposition dishes were added into cages of diapausing adults and collected from the adult cages within the same week. Once removed from the adult cage, oviposition dishes were incubated for 11 d at 25 °C (egg age ranged from 12 to 18 d). The oviposition dishes were then transferred to primary rearing containers containing 3 d old corn seedlings (see more information below under rearing). After remaining in the primary rearing containers for 7 d, the oviposition dishes were transferred to a second primary rearing container and allowed to remain for 7 d. Larvae hatching from the oviposition dishes for the 14 d period (or within 19–32 days after oviposition) were able to leave the oviposition dish and had a food source available. These primary rearing containers then proceeded through the normal rearing pipeline outlined below. This process was continued using diapausing egg dishes until sufficient adults (250 ♂ : 500 ♀ per generation) were obtained from the selection process to start a second cycle of selection using eggs produced from non-diapausing females after generation five.

Egg hatching experiments were performed to identify the percentage of eggs that had hatched, were dead, or were exhibiting diapause starting on the third generation of selection for the non-diapause trait. For each generation evaluated, three subsets of northern corn rootworm eggs (100 eggs per subset) were collected from different oviposition dishes (egg age ranged from 1–8 d). For each subset, the eggs were transferred onto a 100 × 15 mm polystyrene Petri dish containing a moist filter paper. Parafilm® was used to seal the Petri dish. The egg dishes were incubated for 14 d at 25 °C and 60% RH. After 3 d post incubation, egg hatch and dead eggs were recorded every day, and these eggs and emerging larvae were then removed from the egg dishes. Remaining diapause eggs (eggs in good condition, and not hatched or dead) were recorded at 14 d. The filter paper was checked daily and was moistened as needed to ensure that the paper was not dried or saturated.

### Rearing techniques for both diapausing and non-diapausing strains

### Egg storage, egg handling and preparation for hatching

#### Diapausing colony

*Egg storage* Prior to cold storage, eggs were incubated for 14 d at 25 °C to allow for prediapause embryonic development. After 14 d, eggs were washed from soil with cold water (~ 7–9 °C) through a 12-mesh sieve (1.68-mm openings) placed on top of a 60-mesh sieve (0.25-mm openings). Any remaining soil clumps were carefully broken and worked through the 12-mesh sieve. Any remaining clods, stones, or insect debris was discarded. Particles captured in the 60 mesh sieve were rinsed into a container and poured into a 2 L container with saturated magnesium sulfate solution^44^. Soil particles sank in the solution, while eggs/organic debris floated. The egg/organic debris portion was decanted into a 60-mesh sieve and rinsed to remove the magnesium sulfate. The egg/organic debris portion was then suspended in water, where intact eggs sank and organic debris that floated was easily removed. Eggs in water were moved to a graduated cylinder for quantification. The graduated cylinder was gently tapped on the bench surface to compact eggs and measure egg volume. For western and northern corn rootworms, 1 ml of eggs is ~ 10,000 eggs^45^. Eggs then were collected using a 7 ml disposable pipette and placed in 100 × 25 mm polystyrene Petri dishes and covered with ~ 7 mm depth of 80 mesh (0.177 mm) sifted sandy loam soil. Petri dishes were then stacked inside a plastic tube with a screen top, labeled and stored at 8 °C. Eggs were stored for a minimum of five months to break diapause and synchronize hatch.

*Egg recovery* After removing from cold storage, diapausing northern corn rootworm eggs were recovered from the storage dish soil by washing them through a 60-mesh sieve with cold water (~ 7–9 °C). Eggs were retained in the sieve while soil passed through. Eggs were washed with water and counted using the same method as previously described.

*Inoculation container* After removal from oviposition soil, eggs were placed in inoculation containers (Solo^®^ soufflé plastic portion containers; 44 ml; P150N with lids). To facilitate egg quantification, eggs were re-suspended in cold (~ 7–9 °C) 0.15% agar solution (Bacto™ Agar, Fisher Scientific, Pittsburgh, PA). Egg density/ml of agar solution was determined by counting eggs in 10–1 ml aliquots collected using a 10 ml serological pipette. The egg density was then adjusted to 40–60 eggs/ml. Soil was added to each inoculation container: 15 g of dry soil (clay loam to sandy clay loam that is free of pesticide residue and slightly alkaline is preferred) that was air dried thoroughly but not oven dried or autoclaved. Soil was leveled, then 6 ml of northern corn rootworm egg solution (300–360 eggs/container) were added to the soil surface and an additional 15 g of dry soil was spread evenly over the eggs. Inoculation containers were sealed with snap lids, labeled, and stored at 8 °C until needed. When eggs were needed, the desired number of inoculation containers were removed from cold storage and incubated at 25 °C and 60% RH in complete darkness for 11 d in order to promote egg hatch and facilitate eclosion of neonate larvae.

#### Non-diapausing colony

After harvesting, the eggs (egg age 1–8 d old) in oviposition egg dishes were washed from soil with tap water (~ 25 °C) and the number of eggs was quantified. The washing and quantification procedure was similar as for diapausing colony, except for the use of tap water. Subsequently, eggs were added directly to primary rearing containers by pipetting 0.06 ml of egg solution in tap water per container (~ 600 eggs per container) (as described below). Earliest hatching was not anticipated for 3–4 days which allowed the corn time to germinate and begin growing providing food for the neonate larvae.

### Larval stages

#### Primary containers

Primary containers consisted of a 13 × 11 × 7 cm disposable plastic container (Pactiv Showcase^®^ deli containers; 945 ml; Lake Forest, IL; YCI86032). Prior to use in primary containers, corn seed was (1) washed with tap water with a few drops of Ivory detergent added, (2) washed with a bleach solution (10% Clorox Regular Bleach, Clorox, Oakland, CA), (3) rinsed with tap water, and (4) soaked overnight in tap water. Approximately 50 pre-soaked corn seeds (Viking 42–92 conventional untreated, Albert Lea Seed, Albert Lea, MN) were placed in the bottom of the container, ~ 60 ml of water was added to the corn seed and the seed was covered evenly with 200 g (~ 175 ml) of dry soil (same as above). For the diapausing colony, the soil from one egg inoculation container was placed onto the surface of the soil and the container was sealed with the snap on lid, whereas the eggs, for non-diapausing colony, were pipetted directly into the primary container at a volume of 0.06 ml of eggs solution in tap water per container (~ 600 eggs per container) prior to the addition of the pre-soaked corn. Primary containers were incubated for 14 d at 25 °C and 60% RH in complete darkness. Eggs began hatching after ~ 4 d, and larvae migrated to the germinating corn seed mat.

#### Secondary containers

Secondary containers were planted 7 d after primary containers. The secondary containers consisted of a 34 × 25 × 10 cm plastic box (Rubbermaid® Cold Food Pan; 7.5 L, Huntersville, NC; 124P). Pre-soaked corn seed (100 mL) was prepared as described in Primary Containers and was then placed in the bottom of the container, covered with 2000 g (~ 1.5 L) of dry soil and spread evenly, and followed by an addition of ~ 400 ml of water to the surface of the soil. The secondary containers were incubated for 7 d at 25 °C and 60% RH in complete darkness, after which they were infested with rootworm larvae by inverting two primary containers onto the surface of the soil. Secondary container lids were modified for ventilation prior to use (Rubbbermaid^®^ Cold Food Pan lid; FG128P23CLR). Nine 1-cm holes were drilled into the lid. Holes were then covered with 18 mesh (1 mm) nylon window screening and sealed with hot glue. The secondary container lids were then placed upside down (to form a dome so there was enough space for the primary containers) and secured with two binder clips (Staples^®^ binder clips; medium; Framingham, MA, 10668-CC). The secondary containers were incubated for an additional 11 d at 25 °C and 60% RH in complete darkness. Larvae from the primary container gradually moved to fresh roots in the secondary container to complete development and pupate.

### Adult collection

#### Emergence containers

**Figure 4:**
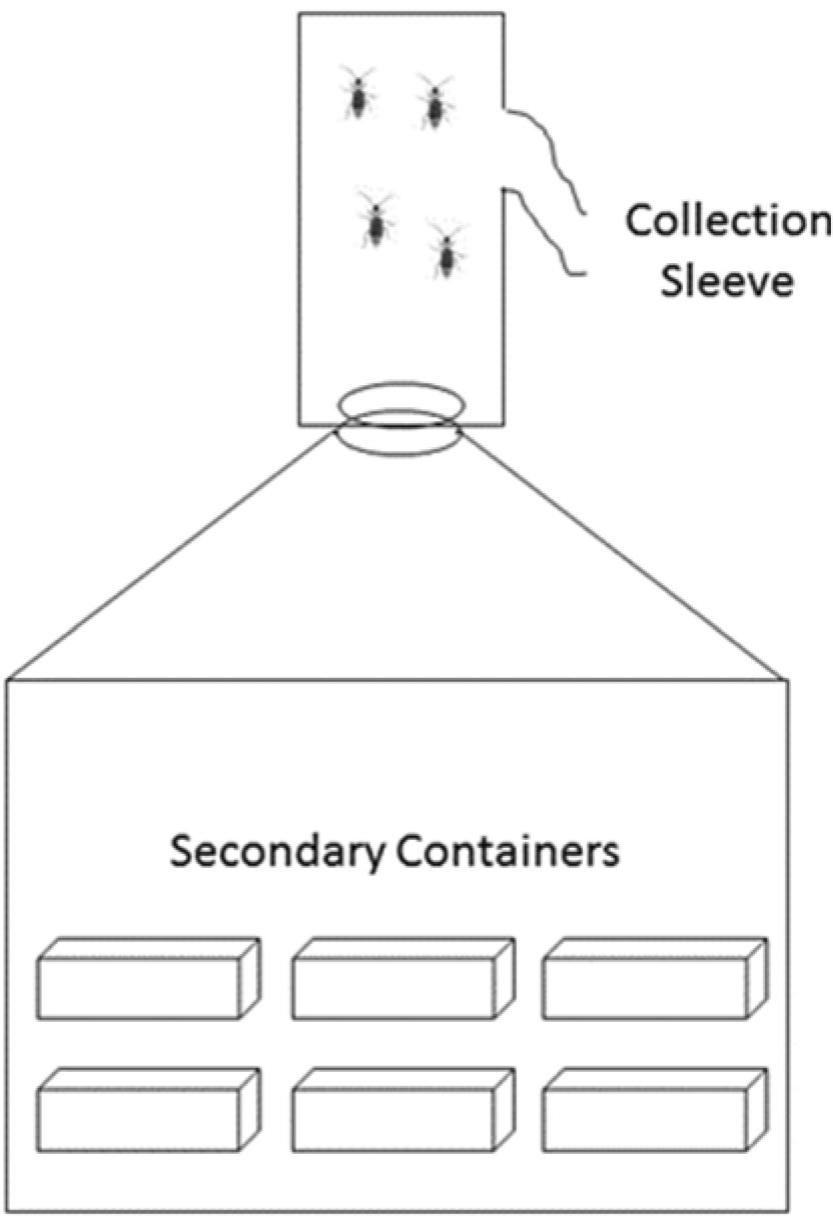
Adult emergence container.

Secondary containers were ready for movement to beetle emergence containers 11 d after infestation with primary containers. Lids were removed from the containers and corn plants were trimmed close enough to the soil to minimize re-growth. We did not pull up the roots or disturb the soil to prevent damaging the pupae. Emergence containers consisted of two parts, a bottom portion where the secondary containers were placed, and a top portion where the adults were eventually collected (Fig. 4). The bottom portion was made of aluminum metal and sealed to prevent adult escape and to prevent light from reaching the secondary containers. The lid for the bottom portion was also made of aluminum metal and was pyramid-shaped to encourage adult beetles to move upwards into the adult collection portion. The adult collection portion was also made of galvanized metal, but with one side replaced with Plexiglas to allow light to enter, thereby encouraging adult movement upward. There was also a collection sleeve in one side of the adult collection portion so that adults were easily collected. Because emerging adults were collected once a week, a small bundle of corn seedlings (~ 7–14 d old), artificial diet^42,43,46^, an agar water source, and a small section of squash [e.g., zucchini (*Cucurbita pepo* L.)] were placed in the adult collection portion. Emergence containers were maintained at 25 °C and 60% RH in complete light.

#### Adult emergence

**Figure 5:**
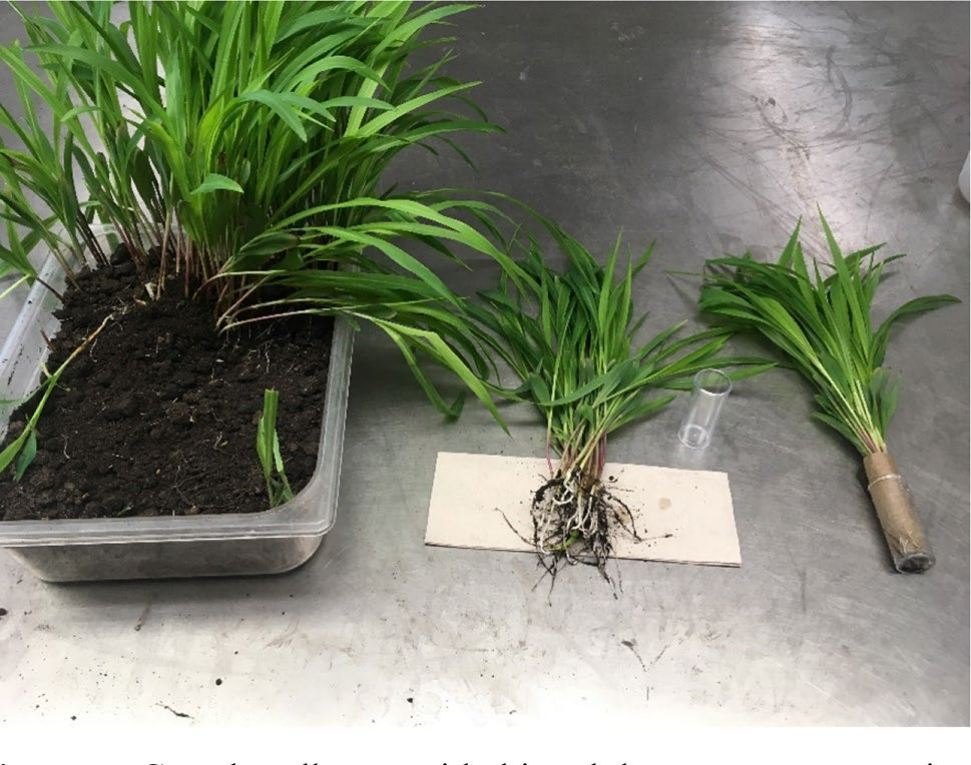
Corn bundles provided in adult emergence container.

**Figure 6:**
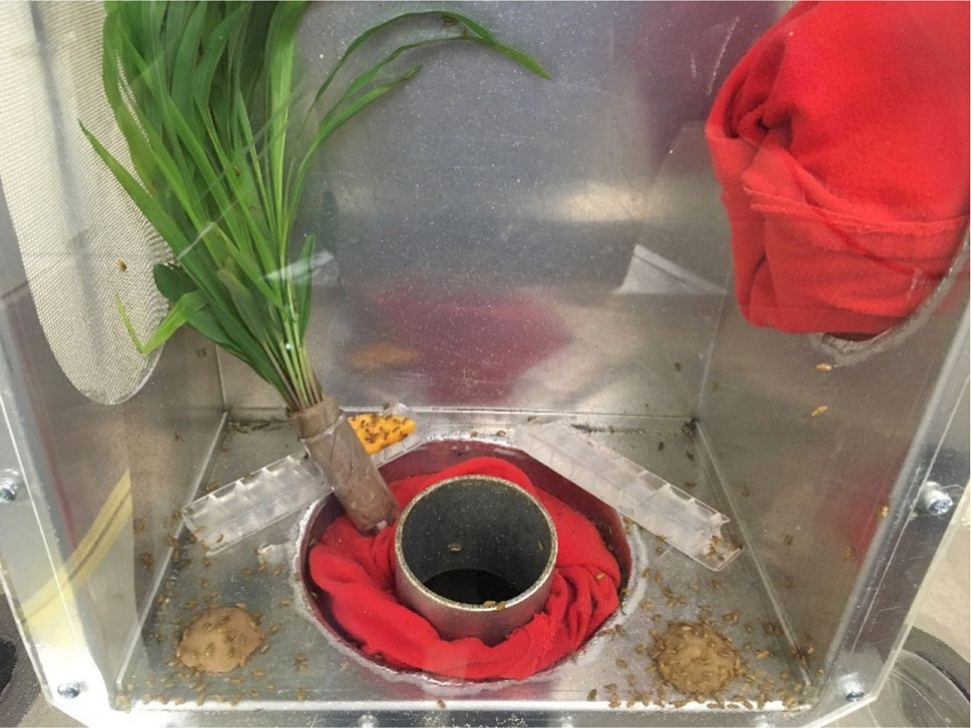
Corn bundle, squash, diet, and water source provided in adult emergence container.

Adults began emerging within 3–10 d after placing in emergence containers. Adults were collected once per week. Food and water (as described above) were provided in the adult collection portion to ensure insects remained healthy until being collected and transferred to adult rearing cages (Figs. 5 and 6). Adult beetles were aspirated from the collection portion, sexed, and added to the rearing cages. Antennal length was the primary characteristic used to differentiate sex^6,47^.

### Adult rearing

#### Cages

Adult cages consisted of a 30.5 × 60 cm wood frame covered with 18 mesh (1 mm) nylon window screening. One side of the cage had a collection sleeve to allow access. Adult cages were maintained at 25 °C and 60% RH under 14:10 L:D.

#### Populating cages

Adult populations were maintained at 250:500 male:female ratio. Because adults were collected once per week, adults were between 1 and 7 d old at the time of collection. Males and females were added to the rearing cage at the time of the collection, and an oviposition dish added to the cage. Cages were maintained for one month before being terminated with a total of up to 30 d of egg collection. Sometimes, due to mortality and a decrease in egg production, adult cages were not maintained beyond 14 d.

#### Artificial diet and water

Adult beetles were fed artificial diet^42,43,46^. Water was provided to adult beetles in the form of 6% agar cubes.

#### Oviposition dishes

**Figure 7:**
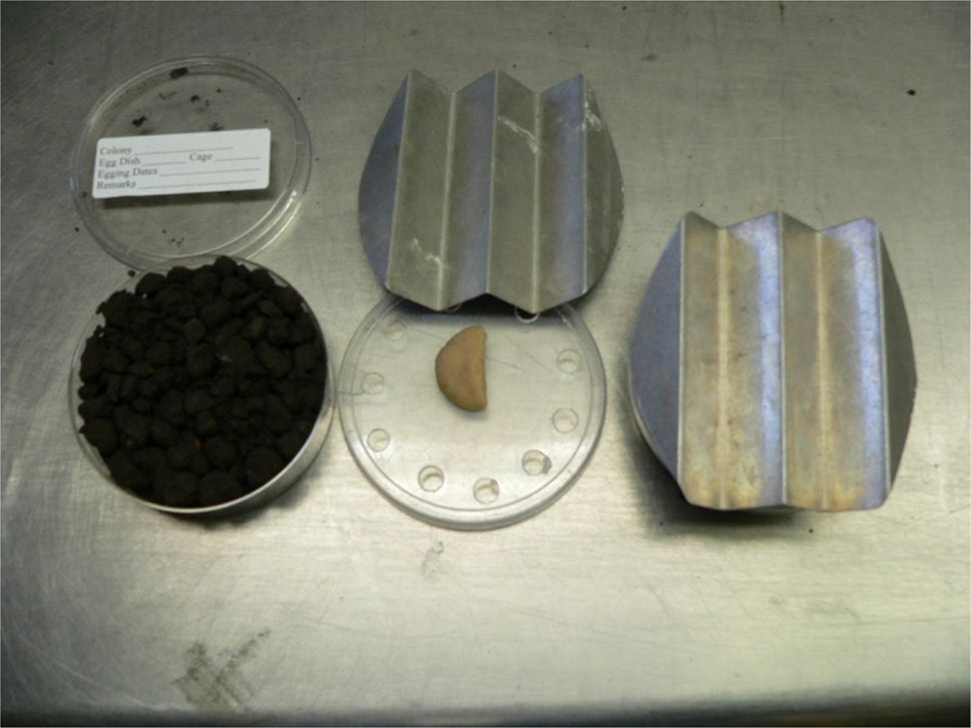
Optimized soil surface for oviposition plates.

An oviposition dish consisted of a 100 × 25 mm polystyrene Petri dish with ~ 35 ml of 80 mesh sifted soil in the bottom covered with 6.4–12.4 ml of dry soil clod (~ 1 cm in diameter). Approximately 30 ml of tap water was then added to the soil and the Petri dish was shaken/tapped to level the soil surface. Any excess water on the surface of the soil was absorbed by the addition of more soil (5–10 ml). The Petri dish was then covered with a lid with 10 7-mm holes drilled around the edge, followed by an addition of an artificial diet slice on top of the Petri dish. Next, the oviposition dish was covered with a pleated piece of aluminum to darken the dish and thereby facilitating oviposition (Fig. 7). The exact size and pleat width of the aluminum sheet varied slightly, but was large enough to cover the Petri dish (Fig. 7). Obtaining optimal soil moisture for oviposition will require some trial-and-error testing, depending on individual soil types. Soil should be firm with well-defined clods.

#### Maintenance

Adult cages were cleaned twice each week. Cage cleaning involved vacuuming the cages to remove dead insects, frass, and loose artificial diet. Artificial diet and water sources were replaced at this time as well. Oviposition dishes were changed during cage maintenance, though only once per week. Oviposition dish replacement was performed in a three-step process. First, the original oviposition dish was uncovered but allowed to remain in the cage for 24 h. Second, the new oviposition dish was added to the cage and covered with lid/diet/aluminum as described above. Lastly, the original oviposition dish was removed 24 h later. This process ensured minimal disturbance of the insects and provided time for adults to move out of the week-old dish into the new dish. After removal from the adult cage, any dead insects were removed from the surface of the oviposition dish, the soil surface was moistened and lightly covered with 80 mesh soil, and the original Petri dish lid replaced before being moved to storage.

### Statistical analyses

Adult emergence from eggs hatched within 19–32 days post oviposition without cold exposure was used as an indication of the development of the non-diapausing strain of northern corn rootworm. For each generation of the selection, several oviposition (4–13 dishes) egg dishes were used to obtain sufficient adults for next generations. Each oviposition dish was considered as a replication. The eggs in each oviposition dish (replicate) were quantitated to calculate total eggs used for each time point. Percentages of adult emergence from eggs for each generation were obtained by dividing the number of adults emerged by the total eggs used and multiplying by 100. For the egg hatching experiments, each subset of northern corn rootworm eggs was used as a replication. Egg hatch, dead eggs, and (diapause) eggs remaining for each generation were expressed as percentages by dividing the number of hatched eggs, dead eggs, and eggs remaining in diapause by the total eggs used and multiplying by 100. All experiments were analyzed as a completely randomized design using PROC MIXED in SAS. Percent variables were arcsine square-root transformed prior to the analysis to meet assumptions of normality and homoscedasticity, while untransformed data were presented as mean ± SEM. Differences between treatments were determined using Fisher’s least significant difference (LSD) at *P* < 0.05.

## Data Availability

All pertinent data are found in the figures and tables. Requests for data and additional information should be submitted to the corresponding author.
